# Fetus in the Abdominal Cavity After Uterine Rupture in a Primigravida Post-Adenomyosis Enucleation

**DOI:** 10.3390/diagnostics14222470

**Published:** 2024-11-05

**Authors:** Saki Kamata, Hanano Ando, Erina Matsuda, Aiko Aoki, Atsushi Komatsu, Kei Kawana

**Affiliations:** Department of Obstetrics and Gynecology, Nihon University School of Medicine, Tokyo 173-8610, Japan; kamata.saki@nihon-u.ac.jp (S.K.); ando.hanano@nihon-u.ac.jp (H.A.); matsuda.erina@nihon-u.ac.jp (E.M.); aoki.aiko@nihon-u.ac.jp (A.A.); komatsu.atsushi@nihon-u.ac.jp (A.K.)

**Keywords:** uterine rupture, magnetic resonance imaging, the previous surgical scar, adenomyosis enucleation

## Abstract

A 35-year-old primigravida with a history of adenomyosis enucleation was diagnosed with abnormal fetal position at 25 weeks of gestation. The patient presented with normal vital signs and no symptoms. A cardiotocogram and transabdominal ultrasound revealed a healthy fetus, normal amniotic fluid volume, and no intra-abdominal bleeding. Pelvic magnetic resonance imaging (MRI) indicated a ruptured muscular layer of the uterine fundus, with the fetus completely prolapsed into the abdominal cavity. An emergency cesarean section was performed, during which the fetus was found wrapped within the amniotic membrane in the abdominal cavity. The uterus exhibited extensive tearing along the line of the previous surgical scar; however, no hemorrhage was observed. In this case, despite uterine rupture, blood flow through the umbilical cord from the placenta in the uterus resulted in the survival of the fetus. In addition, MRI was essential in determining the appropriate timing to save the fetus.

Pregnancy and live birth rates are low in patients with adenomyosis, negatively impacting fertility outcomes [1,2]. Although the number of cases of adenomyosis enucleation is low, the efficacy of enucleation in combination with a gonadotropin hormone-releasing hormone (GnRH) agonist or with a GnRH agonist alone has been compared, indicating that surgical treatment can increase spontaneous pregnancy rates [2]. Furthermore, enucleation of adenomyosis may also reduce adverse pregnancy outcomes associated with the condition, such as preterm pre-labor rupture of membranes, preeclampsia, and small-for-gestational-age infants [3]. However, complications such as uterine rupture and adherent placenta should also be noted in pregnancies following the enucleation of uterine adenomyosis. The incidence of these complications is higher in cases of a scarred uterus, such as after cesarean section, myoma, or enucleation of adenomyosis than in cases of an unscarred uterus [4,5]. Specifically, the risk of uterine rupture after enucleation of adenomyosis is reported to be 6.0%, which is higher than that of other surgeries [6]. There are no clear standards for the duration of postoperative contraception; however, the risk of uterine rupture is two to three times greater within 24 months of cesarean section. In addition, while the resumption of blood flow at the wound site occurs within 6 months in 80% of cases, it may take more than 2 years if the myometrium of the entire circumferential region is removed or if electrocautery is used [6]. Typical symptoms of uterine rupture include abnormal fetal heartbeat, pain, and vaginal bleeding. However, characteristic signs predictive of uterine rupture have not been reported, and a combination of these symptoms is uncommon [7,8].

Although uterine rupture is rare, it remains a significant concern owing to the poor fetal survival rate when evacuation occurs in the abdominal cavity. In this report, we present a case of asymptomatic uterine rupture that was diagnosed using MRI.

A 35-year-old primigravida was diagnosed with abnormal fetal position when the fetus was visualized in the upper abdomen on transabdominal ultrasonography during a routine antenatal checkup at 25 weeks of gestation. She had undergone type II open uterine adenomyosis enucleation with electrocautery, a procedure for circumferential adenomyosis of the uterine fundus, 2 years earlier and subsequently conceived through intracytoplasmic sperm injection. The patient’s vital signs were normal, and she had no abdominal pain. Cardiotocography and transabdominal ultrasonography revealed a healthy fetus with normal amniotic fluid volume and no intra-abdominal bleeding. There was no genital bleeding, and the fetus could not be visualized on transvaginal ultrasonography. The placenta covered the endocervical opening. No uterine contractions were observed. Based on ultrasound alone, it was difficult to diagnose the uterine rupture and determine whether a cesarean section should be performed. Pelvic MRI revealed a ruptured muscular layer of the uterine fundus, which caused the fetus to prolapse completely into the abdominal cavity. The placenta was attached to the lower uterine body, with no evidence of placental abruption or hematoma. The fetus was encased in the omentum, but no amniotic fluid leak was observed in the abdominal cavity (Figure 1 and Figure 2). An emergency cesarean section was performed, during which the uterus exhibited tearing along with a previous surgical scar; however, no hemorrhage was observed (Figure 3). Pathological examination revealed a fibrotic area approximately 5 mm thick at the rupture site, suggesting that the area of adenomyosis was the rupture site. Despite uterine rupture, blood flow through the umbilical cord from the placenta resulted in fetal survival. The baby girl weighed 912 g (appropriate for gestational age at 25 weeks) and had an Apgar score of 3 for 1 min and 6 for 5 min. The mother was discharged 4 days after surgery. In addition, after 4 months in the neonatal intensive care unit owing to premature delivery, the infant was discharged in healthy condition. At her 3-year checkup, she exhibited normal growth and development.

Adenomyosis enucleation is reportedly associated with an increased pregnancy rate [2]; however, the risk of uterine rupture is high, and it is difficult to diagnose cases of asymptomatic uterine rupture. In the present case, the only indication of uterine rupture was the visualization of the fetus in the mother’s upper abdomen. Although fetal detachment from the pelvic inlet and position shift caused by uterine rupture can be detected on ultrasound, identifying the exact rupture site and placental location remains challenging [9]. In cases of silent uterine rupture, there is no risk of maternal death due to hemorrhage; however, the fetus’s survival depends on the location of the placenta and the presence of abruption. These factors can be assessed using MRI [10].

The present case highlights the importance of risk management and diagnosis in pregnancies following adenomyosis enucleation, confirming that accurate diagnosis of uterine rupture using MRI contributes to improved fetal survival.

## Figures and Tables

**Figure 1:**
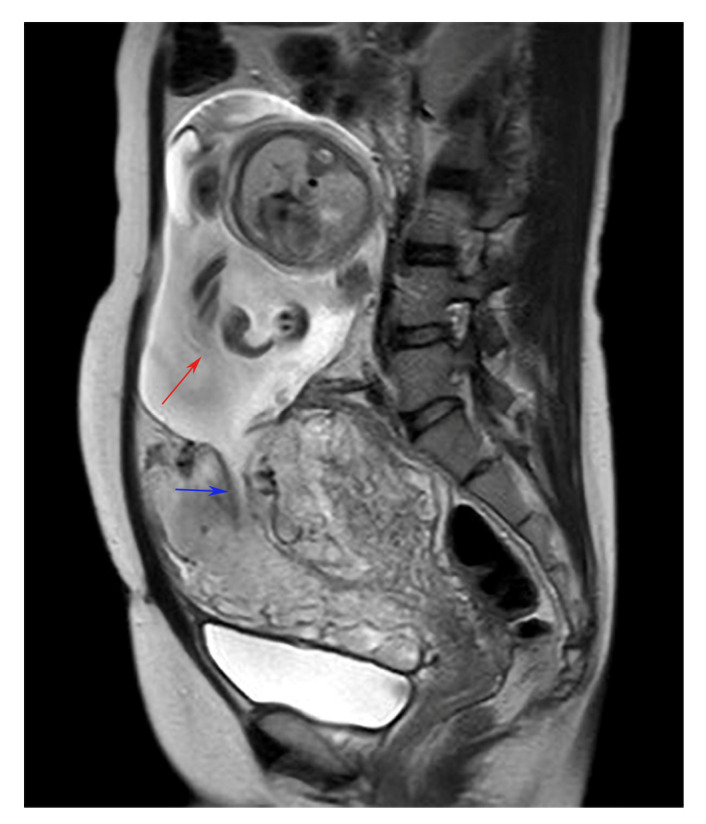
Pelvic MRI (sagittal) showing that the muscular layer of the uterine fundus was ruptured and the fetus had completely prolapsed into the abdominal cavity. The fetus is indicated by the red arrow, and the wound in the uterus is indicated by the blue arrow.

**Figure 2:**
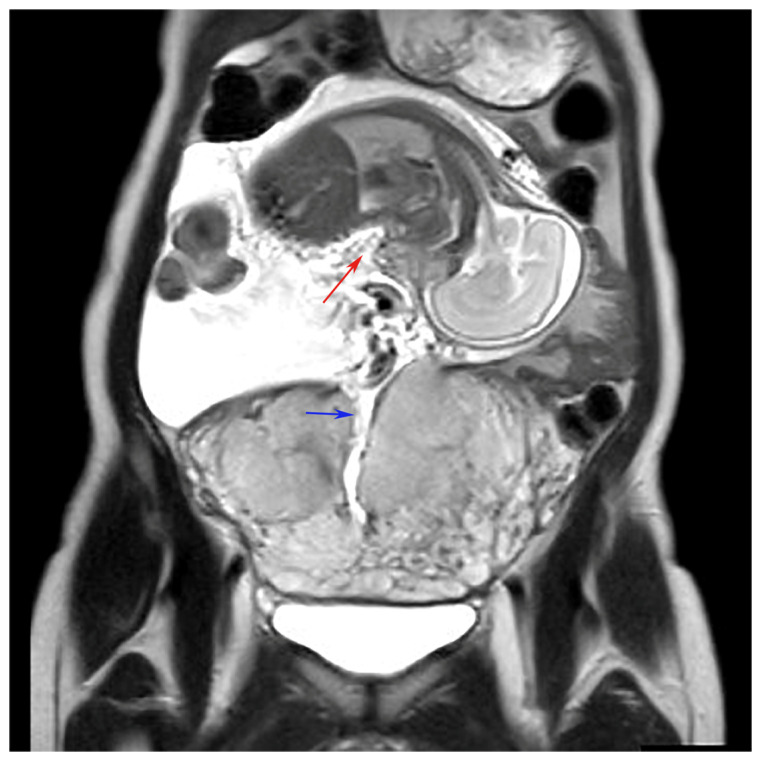
Pelvic MRI (coronal) showing that the muscular layer of the uterine fundus was ruptured and the fetus had completely prolapsed into the abdominal cavity. The fetus is indicated by the red arrow, and the wound in the uterus is indicated by the blue arrow.

**Figure 3:**
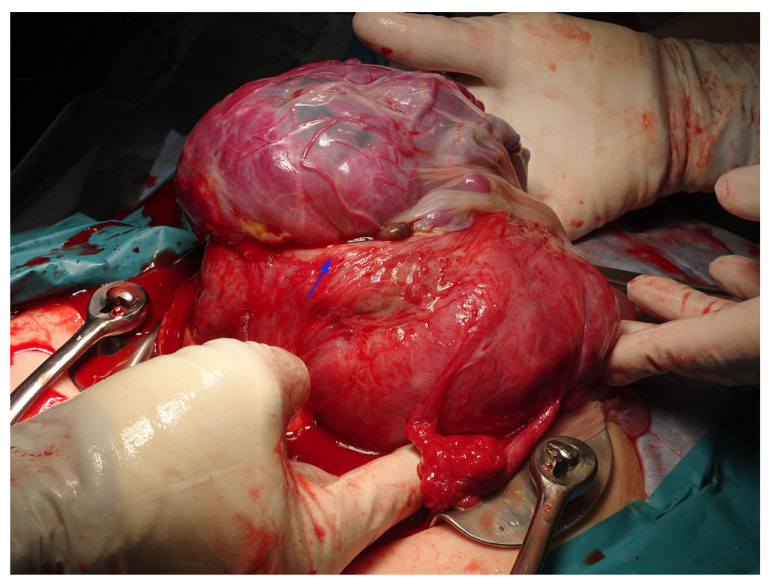
Intra-abdominal findings during an emergency cesarean section reveal a tear in the myometrium along the previous wound at the time of myomectomy. The wound in the uterus is indicated by the blue arrow.

## Data Availability

No new data were created or analyzed in this study.
